# Somali women and their pregnancy outcomes postmigration: data from six receiving countries

**DOI:** 10.1111/j.1471-0528.2008.01942.x

**Published:** 2008-12

**Authors:** R Small, A Gagnon, M Gissler, J Zeitlin, M Bennis, RH Glazier, E Haelterman, G Martens, S McDermott, M Urquia, S Vangen

**Affiliations:** a Mother & Child Health Research, La Trobe UniversityMelbourne, Victoria, Australia; b McGill University and McGill University Health CentreMontreal, Quebec, Canada; c STAKES (National Research and Development Centre for Welfare and Health)Helsinki, Finland; d Nordic School of Public HealthGothenburg, Sweden; e INSERM, UMR S149, Epidemiological Research Unit on Perinatal and Women’s HealthParis, France; f Université Pierre et Marie Curie-Paris 6Paris, France; g Centre for Epidemiology, Socialstyrelsen (National Board of Health and Welfare)Stockholm, Sweden; h Institute for Clinical Evaluative SciencesToronto, Ontario, Canada; i Observatoire de la Santé et du Social de Bruxelles-CapitaleBruxelles, Belgium; j Service de Pédiatrie, CHU Saint PierreBrussels, Belgium; k Studiecentrum voor Perinatale Epidemiologie (SPE)Flanders, Belgium; l Public Health Agency of CanadaOttawa, Ontario, Canada; m University of TorontoToronto, Ontario, Canada; n National Resource Center for Women’s Health, RikshospitaletOslo, Norway

**Keywords:** Caesarean section, pregnancy outcomes, migration, Somali, stillbirth

## Abstract

**Objective:**

This study aimed to investigate pregnancy outcomes in Somali-born women compared with those women born in each of the six receiving countries: Australia, Belgium, Canada, Finland, Norway and Sweden.

**Design:**

Meta-analyses of routinely collected data on confinements and births.

**Setting:**

National or regional perinatal datasets spanning 3–6 years between 1997 and 2004 from six countries.

**Sample:**

A total of 10 431 Somali-born women and 2 168 891 receiving country-born women.

**Methods:**

Meta-analyses to compare outcomes for Somali-born and receiving country-born women across the six countries.

**Main outcome measures:**

Events of labour (induction, epidural use and proportion of women using no analgesia), mode of birth (spontaneous vaginal birth, operative vaginal birth and caesarean section) and infant outcomes (preterm birth, birthweight, Apgar at 5 minutes, stillbirths and neonatal deaths).

**Results:**

Compared with receiving country-born women, Somali-born women were less likely to give birth preterm (pooled OR 0.72, 95% CI 0.64–0.81) or to have infants of low birthweight (pooled OR 0.89, 95% CI 0.82–0.98), but there was an excess of caesarean sections, particularly in first births (pooled OR 1.41, 95% CI 1.25–1.59) and an excess of stillbirths (pooled OR 1.86, 95% CI 1.38–2.51).

**Conclusions:**

This analysis has identified a number of disparities in outcomes between Somali-born women and their receiving country counterparts. The disparities are not readily explained and they raise concerns about the provision of maternity care for Somali women postmigration. Review of maternity care practices followed by implementation and careful evaluation of strategies to improve both care and outcomes for Somali women is needed.

## Introduction

Somali women have fled their strife-torn homeland since the early 1990s. Many remain in refugee camps in Africa, but large numbers have also subsequently settled in developed countries in Europe, in the USA and Canada, and in Australia. Three studies of Somali women’s pregnancy outcomes postmigration have been undertaken – one in the USA^1^, one in Norway^2^ and one small study in the UK.^3^ Two studies of women from the Horn of Africa (including Somalia) have also been reported from Sweden^4^ and Norway.^5^ These have identified a number of disparities in comparison to outcomes in the receiving populations. Higher rates of anaemia^1^ and gestational diabetes^1^,^5^ have been found in Somali women, but lower rates of hypertension and pre-eclampsia.^5^ There have been mixed findings with respect to rates of induction in Somali women, with one study reporting lower rates,^1^ one higher^2^ and a third finding no differences.^3^ Lower use of pain relief in labour^1^,^4^ and lower rates of epidural use, 1–5 as well as higher rates of caesarean section among Somali women have been found.^1^,^2^,^4^,^5^ More perineal trauma has been reported for Somali women,^1^,^2^ and postpartum haemorrhages were more common in Somali women in the Norwegian study,^2^ although not in the US study.^1^ Higher rates of stillbirth among Somali women are also apparent,^1^,^2^,^6^ as are 5-minute Apgars of less than 7 in liveborn infants.^1^,^2^

A number of interview studies have also investigated Somali women’s experiences of maternity care after resettlement, identifying concerns about care and highlighting important challenges for achieving the provision of sound, respectful and appropriate maternity care for resettled Somali women.^7^–^10^ Study participants expressed appreciation for the care received, but issues raised included fears about seeking antenatal care in their new homeland; considerable anxiety and fear about interventions, particularly caesarean section; preference for female companion support in labour; difficulties in communication with caregivers because of language problems and lack of access to interpreters; perceptions of poor nursing and medical care; and concerns about the lack of experience of caregivers to deal appropriately with childbirth in women who had undergone traditional genital cutting and infibulation. One Swiss and two Norwegian papers report health provider views and delivery practices for infibulated African women and note that caesarean section was sometimes resorted to, rather than undertaking defibulation to enable women to give birth vaginally.^11^–^13^ One of the Norwegian reports also highlighted the tendency by care providers to over-interpret Somali culture in their care of women, rather than clarify the relevant cultural issues with individual women themselves.^13^ In some cases, this tendency led to caesarean section to ‘protect’ women’s infibulation in the name of culturally sensitive care.

The current study reports data from six countries: Australia, Belgium, Canada, Finland, Norway and Sweden with the aim of investigating pregnancy outcomes for Somali women postmigration compared with those for women born in each of these receiving countries. Country inclusion was facilitated by access to relevant national or regional data sets and by the authors’ participation in an international collaboration of perinatal researchers: Reproductive Outcomes And Migration was first formed in September 2005. A primary purpose of the collaboration has been to determine the feasibility of international comparisons of reproductive outcomes for groups of immigrant and refugee women in resettlement countries and, where this is feasible, to undertake such comparisons to contribute to understanding and improving the care that women receive.

## Methods

### Country data sources

Data were contributed from six countries using national or regional data sets that enabled identification of all births to women born in Somalia over periods of 3–6 years between 1997 and 2004. Three countries provided data from two regions each and three provided national data.

### Description of the data sets

#### Australia

Australian data were obtained for the two largest states (New South Wales and Victoria) where more than half of all births in the country occur and where there are also the largest proportions of births to Somali women. Data on all births and on the health of mothers and babies are routinely collected and reported in both states. Maternal country of birth is an included routine data item. In Victoria, data were obtained from the Perinatal Data Collection Unit in the Victorian Department of Human Services, and in New South Wales (NSW), from the Midwives Data Collection of the NSW Department of Health. For the purposes of the analyses presented in this paper, only data for women admitted for birth as public patients were included, given country of birth disparities in uptake of private health insurance in Australia. The proportions of all Somali-born women who gave birth as public patients during 1999–2003 was 99.1% in NSW and 99.5% in Victoria compared with 67.2% of Australian-born women in NSW and 63.6% in Victoria.

#### Belgium

Data were obtained for two regions – Flanders and Brussels, where routine reporting on births includes completion of three data charts: the obstetric and perinatal data chart and the neonatal data chart, both completed by maternity care providers; and the socio-economic chart completed by the parents on registration of the birth.

#### Canada

Data were provided for two provinces, Ontario and Quebec, using Citizenship and Immigration data linked to administrative hospital records through provincial health registries. Births to women outside of hospital are therefore not included. Given the need to use linked administrative data, a number of relevant variables were also not available, including marital status at the time of delivery, parity, birthweight, gestation, Apgar scores and perinatal or maternal death. Comparison receiving population data included Canadian-born women *and* also women who had migrated to Canada before 1985 matched on age and region of residence. Given Somali migration patterns to Canada, this is unlikely to mean that Somali-born women are included in the receiving population data.

#### Finland

Data for birth-related variables were from the 1999–2001 national Finnish Medical Birth Register at STAKES (National Research and Development Centre for Welfare and Health), which was linked to the Population Register for maternal data on country of birth, native language and citizenship.

#### Norway

Norwegian data were provided from the Medical Birth Registry of Norway, a national database of all births in Norway, which includes a wide range of maternal and neonatal data for each birth, and maternal country of birth was identified through linkage with national census data from Statistics Norway.

#### Sweden

Data from Sweden were provided from the Swedish Medical Birth Register, the national database of pregnancies and births in Sweden. Data are provided to the register by all Swedish maternity services and data were linked to Statistics Sweden to include information on maternal country of birth.

### Common variables/indicators

We derived a common variable set from the range of maternal, pregnancy, labour, birth and infant outcome data collected in each country. Items that could be included in the analysis were maternal age, parity, marital status, induction of labour, analgesia, epidural use, mode of birth, preterm birth (<37 weeks), birthweight, Apgar at 5 minutes, stillbirths and neonatal deaths (<7 and/or <28 days). Four data sets had data on all 13 variables (Australia–NSW and Victoria; Belgium–Flanders and Norway). The Belgium-Brussels data set had all but parity, Sweden had all but analgesia, Finland had all but Apgar, and five of the 13 variables were available in the two Canadian data sets.

### Statistical analysis

Within-country comparisons of maternal age, parity and marital status between Somali-born and receiving country-born women were assessed using StatCalc in EpiInfo (ORs and 95% CIs). Meta-analyses pooling available data for events of labour and birth (induction of labour, no analgesia, epidural use and caesarean section) and infant outcomes (preterm birth, birthweight <2500 g, Apgar ≤7, stillbirths and neonatal deaths) were carried out using a random effects model in Review Manager, version 4.2.10. Meta-analyses for caesarean section were stratified by parity because of the strong association of caesarean section in a first birth with subsequent mode of birth. The impact of statistical heterogeneity was assessed using the *I*^2^ statistic which describes the percentage of the variability in effect estimates due to heterogeneity, with a value greater than 50% considered to indicate substantial heterogeneity.^14^

## Results

### Data set and sample characteristics

Table 1 presents the characteristics of the data sets and of the included samples of Somali-born and receiving country-born women. The number of Somali-born women in each data set varies considerably from just 72 (0.3%) in the Belgium-Brussels data set to 3450 (0.8%) in the Swedish data set.

**Table 1:** Data set and sample characteristics

Country	Period	Somali-born women (%)					Receiving country-born women (%)				
		*n*	Aged <20*	Aged 35****	Nulliparous	No partner	*n*	Aged <20*	Aged 35****	Nulliparous	No partner
Australia—NSW	1999–2003	227	3.5***	15.4**	26.4***	9.3***	204,675	7.8	11.2	40.7	32.2
Australia—Victoria	1999–2003	897	2.2***	14.0**	20.5***	18.8**	149,479	5.4	13.1	41.1	20.0
Belgium—Brussels	1998–2004	72	5.6**	14. 6**	n/a	45.1****	25,371	1.9	11.8	46.6	28.2
Belgium—Flanders	2000–2004	137	5.8****	15.3**	32.1***	45.5****	306,019	2.3	18.0	n/a	16.0
Canada—Quebec	1997–2000	109	1.2***	17.7***	n/a	n/a	208,558	4.5	19.7	n/a	n/a
Canada—Ontario	1997–2000	2,418	6.4***	13.8**	n/a	n/a	354,377	13.4	14.6	n/a	n/a
Finland	1999–2001	833	2.5**	13.6***	13.8***	9.4***	158,911	3.0	18.4	41.2	13.5
Norway	1999–2003	2,288	3.1**	16.4***	22.0***	25.3****	310,923	2.5	15.1	40.7	10.9
Sweden	1999–2003	3,450	2.0**	16.6**	17.1***	30.0****	450,578	1.9	17.7	44.1	5.2
Total		10,431					2,168,891				

* Aged <22 for Canada-Quebec n/a = not available.

** No significant differences between Somali-born women and the receiving country-born population.

*** Somali-born women were significantly less likely than the receiving country-born population to be younger/older/nulliparous/have no partner.

**** Somali-born women were significantly more likely than the receiving country-born population to be younger/older/nulliparous/have no partner.

Somali-born women were less likely to be younger than 20 compared with receiving country-born women (reference category 20–34 years), in four of the data sets; there were no significant differences in four; and in just one were they more likely to be younger. In three data sets, Somali-born women were significantly less likely to be older (35 years or more versus 20–34 years) than receiving country-born women; and in six there were no significant differences. Somali-born women were significantly less likely to be nulliparous than receiving country-born women in all six data sets where parity was available. Of the seven datasets with marital status data, Somali-born women were more likely not to have a partner (i.e. were not married or living with a partner) in four data sets; less likely not to have a partner in two; and there were no significant differences in one.

### Labour

**Figure 1:**
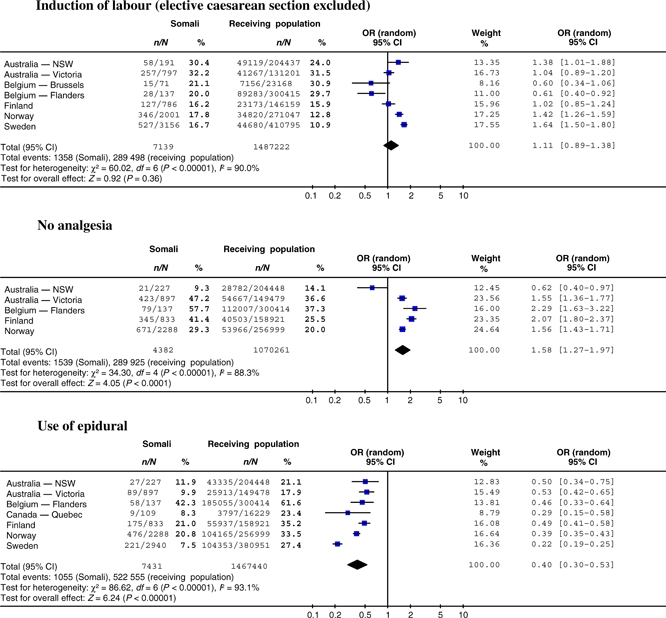
Meta-analyses for events of labour: women born in Somalia compared with women born in receiving countries.

Figure 1 shows both the numbers and proportions of Somali-born and receiving country-born women who experienced induction of labour, used no analgesia, or had an epidural and also the results of the meta-analyses for each of these variables.

Considerable variation between countries in the prevalence of these events/procedures can be seen both among receiving country-born women and among Somali-born women. For the Somali-born women, there are up to two-fold differences seen in the prevalence of induction, six-fold differences in no use of analgesia, and five and a half-fold differences in epidural use between countries. For the receiving country-born women, there are up to three-fold differences in the prevalence of induction between countries, the proportion of women using no analgesia varies by a factor of two and a half, and epidural use also varies by a factor of more than three. These variations in clinical practice between countries are the likely explanation for the statistical heterogeneity seen in the results of the meta-analyses for these variables. The extent of heterogeneity in effect estimates (*I*^2^> 80% for all three) suggests that the pooled odds ratios should be interpreted with caution.

Somali-born women had significantly higher odds of induction compared with receiving country-born women in three of the seven data sets with data on induction. In one data set, Somali-born women had significantly lower odds of induction. In four of the five data sets with information on analgesia use, Somali-born women were significantly more likely than their receiving country-born counterparts to use no analgesia; in the other data set, Somali-born women were significantly less likely to use no analgesia. In all the seven data sets with information on epidural use, Somali-born women were significantly less likely to undergo an epidural than receiving country-born women.

### Mode of birth

**Figure 2:**
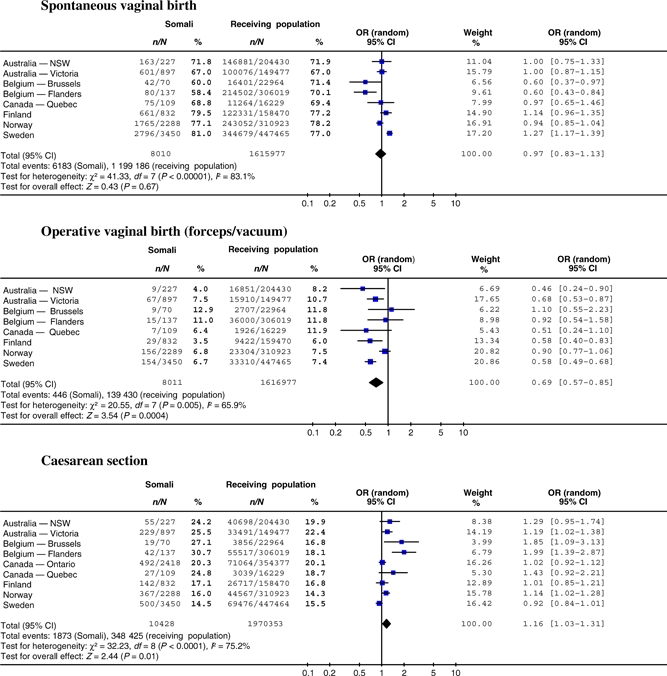
Meta-analyses for mode of birth: women born in Somalia compared with women born in receiving countries.

Figure 2 presents data on mode of birth in three categories: spontaneous vaginal delivery, operative vaginal delivery and caesarean section. Data from Canada-Ontario did not differentiate between operative and spontaneous vaginal birth and have therefore not been included. There is wide variation across the data sets in the prevalence of spontaneous vaginal birth, from 58.4 to 81.0% in Somali-born women and from 67.0 to 78.2% in the receiving country-born populations. In two data sets, Somali-born women were significantly less likely to give birth spontaneously, in one they were more likely to do so. In the other data sets, there were no significant differences. Four of the data sets show significantly lower odds of operative vaginal birth among Somali-born women. Considerable between-country variation is also seen in caesarean section rates, which range between countries from 14.5 to 30.7% in Somali-born women and from 14% to 22.4% in receiving country-born women. In four data sets, Somali-born women had significantly raised odds of caesarean section compared with their receiving country-born counterparts.

**Figure 3:**
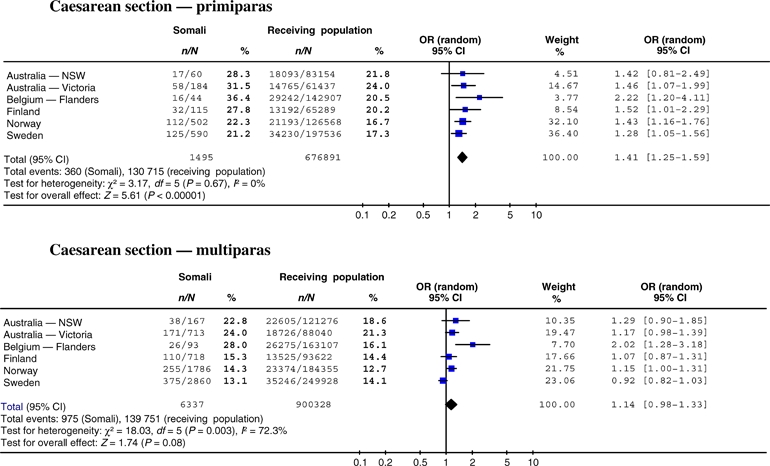
Meta-analyses for caesarean section stratified by parity: women born in Somalia compared with women born in receiving countries.

Heterogeneity in the effect estimates for mode of birth differences means that the pooled estimates should be interpreted with caution. However, Figure 3 shows two meta-analyses describing the findings for caesarean section stratified by parity (primiparous/multiparous). Somali-born women having their first baby had a significantly raised pooled odds ratio of 1.41 (95% CI: 1.25–1.59), with no evidence of heterogeneity (*I*^2^= 0). Multiparas were also at increased risk in two data sets.

### Infant outcomes

**Figure 4:**
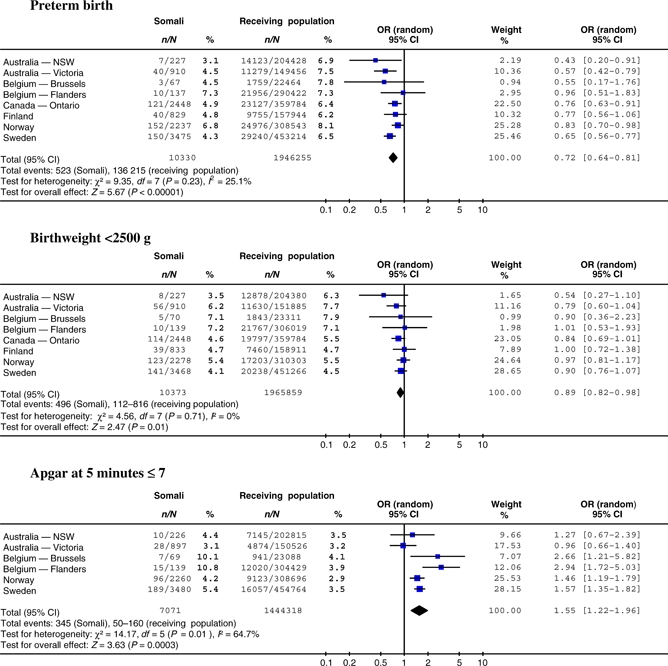
Meta-analyses for preterm birth, birthweight and Apgar scores: births to women born in Somalia compared with women born in receiving countries.

**Figure 5:**
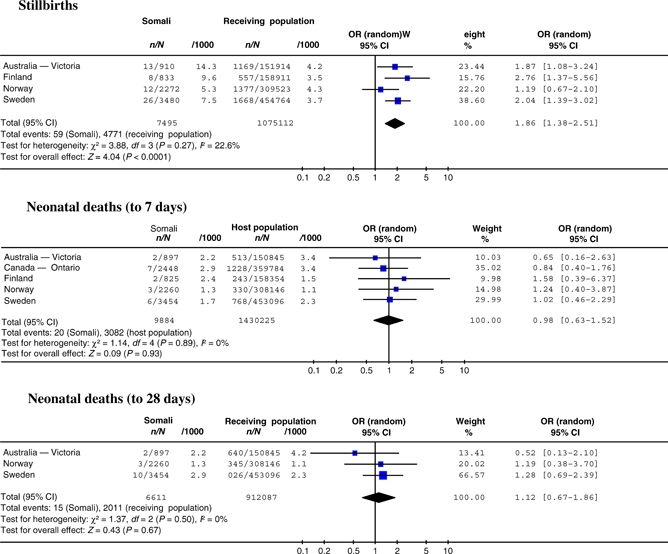
Meta-analyses for stillbirths and neonatal deaths (7 and 28 days): births to women born in Somalia compared with women born in receiving countries.

Data on infant outcomes are presented in Figures 4 and 5, and include preterm birth, birthweight under 2500 g, condition of the newborn (Apgar score at 5 minutes ≤7) and stillbirths and neonatal deaths (to 7 and 28 days).

Infants of Somali-born women were significantly less likely to be preterm than infants of receiving country-born women in the overall pooled estimate, pooled odds ratio 0.72 (95% CI: 0.64–0.81). Similarly, the pooled estimates for birthweight less than 2500 g showed that the infants of Somali-born women were less likely to be of low birthweight than the receiving country-born comparison population infants, pooled odds ratio 0.89 (95% CI: 0.82–0.98). Somewhat in contrast to these findings, infants of Somali-born women were significantly more likely however, to have Apgar scores of ≤7 in four of the six data sets that had data on the condition of the newborn at 5 minutes. The extent of statistical heterogeneity present in the meta-analysis suggests that the pooled odds should be treated with caution.

Figure 5 presents meta-analyses for stillbirths and neonatal deaths for the five countries with available data and where the datasets included sufficient numbers of births to Somali women to make mortality comparisons meaningful (range: 833–3480 births). It is important to note that the gestational age thresholds for births and deaths vary considerably between these five countries. Australia assigns stillbirths and deaths from 20 weeks of gestation (or if gestation is unknown, birthweight of at least 400 g), Canada and Finland from 22 weeks of gestation, Norway from 16 weeks (although data shown are restricted to stillbirths and deaths from 22 weeks) and Sweden from 28 weeks. There are significantly raised odds of stillbirth shown in the pooled analysis, pooled odds ratio 1.86 (95% CI: 1.38–2.51), with little evidence of heterogeneity. The pooled odds for neonatal deaths show no significant differences.

## Discussion

This study represents the largest pooled data set yet assembled of confinements and births to Somali-born women giving birth outside Somalia and we have been able to compare a number of important indicators of perinatal health. The findings show no excess of problems with preterm birth or low birthweight among Somali-born women, but aspects of the delivery process do show cause for concern, particularly the excess in caesarean sections and stillbirths among Somali-born women compared with receiving country-born women.

The study has limitations. As expected,^15^ considerable variation was found between (and within) countries in the information routinely collected about pregnancy and birth. This variability meant that comparisons were only possible for a core set of common indicators, and even then, not for all countries in every instance. Many indicators of potential interest were collected in too few of the data sets to include in our comparisons, such as perineal status and postpartum haemorrhage. For other variables, the data definitions used or the method of reporting—specified variable with tick boxes versus free text reporting—precluded easy comparison (e.g. maternal medical conditions, pregnancy complications and the indications for caesarean section). Despite these problems, what this analysis has shown is that monitoring trends in health care and outcome for Somali women or other specific migrant groups, is possible using routine perinatal and administrative data.

Meta-analyses have been presented for available outcome variables. These require cautious interpretation, given the significant statistical heterogeneity for some indicators, with wide variation in clinical practice evident between, and sometimes within countries. Interpretation of the findings is also necessarily speculative, especially as analyses adjusting for potential confounders were not possible across the data sets. Our findings are broadly consistent, however, with the small number of within-country studies previously reported.

The maternal age data did not demonstrate potentially important risk differences between Somali-born women and women born in the receiving countries. Parity data showed that Somali-born women were more often multiparous than women giving birth in receiving populations. This is likely to be because of larger average family size in the Somali community at home and abroad, and grand multiparity has been suggested as a possible risk factor for some of the poorer outcomes in Somali women in other studies.^1^–^3^ There was considerable variation between countries in whether Somali-born women were more or less likely to be married or partnered. One explanation for this variation may be differences in the refugee intake in the different countries, with some more likely to take unpartnered women with children. Unfortunately, the limited availability of data on refugees in developed nations makes it difficult to find support for this explanation.^16^

Wide variation was evident between countries represented in this study in the use of induction, in rates of analgesia and epidural use, and in rates of caesarean section. In addition, the comparisons between Somali-born and receiving country-born women also produced divergent findings with, for example significantly lower rates of induction in Somali-born women in some countries, and in others, significantly higher rates. However, with one exception, giving birth without any analgesia was consistently more prevalent in Somali-born women, and they were significantly less likely to experience an epidural during labour or birth. Interview studies with Somali women^6^–^11^,^17^ indicate an expressed dislike of interference in the birth process. Language and communication problems may also contribute to differences in the use of pain relief, for example affecting whether women are offered, or feel prepared to undergo an epidural.

The raised caesarean section rates seen here for Somali-born women having their first child is a consistent finding in all published studies comparing Somali-born women with receiving country-born populations. While our findings are not adjusted for potentially relevant risk factors (although they are stratified for parity), population-based studies in three resettlement countries suggest that the excess of caesarean sections in Somali women is unlikely to be fully explained by an excess of clinical risk factors in this population.^1^,^2^,^17^ In a recent study from the USA^1^ comparing Somali-born and US-born women (grouped separately as ‘Blacks’ and ‘Whites’), Somali women having their first child had a two-fold risk of caesarean section after adjustment for gestational age, birthweight and maternal age compared with either ‘black’ or ‘white’ women. An earlier Norwegian study,^4^ reporting combined data for women from the Horn of Africa (Somalia, Eritrea and Ethiopia) found a prevalence of caesarean section in this group of 20.5 compared with 12.4% in Norwegian-born women (an excess crude risk of 8.1%). After adjustment for a range of sociodemographic characteristics and perinatal risk factors, a small excess risk of 2.7% remained. Included in the adjusted perinatal risk factors however, were fetal distress and prolonged labour, two indications for caesarean section known to be ‘applied’ with much variation in clinical practice, and possibly differentially in different population groups. Furthermore, an Australian study that investigated caesarean section rates by maternal country of birth in all women identified as low risk (singleton, term birth, well-grown infant, no cardiac disease, no pre-existing diabetes, hypertension or mental illness, and no reported obstetric complications during the pregnancy) and giving birth to their first baby in the state of Victoria (1999–2003), found the rate of caesarean section for African women (principally from Somalia, Eritrea and Ethiopia) was 28.7% compared with 18.5% for Australian-born women.^17^

If clinical risk factors do not explain the excess of caesarean sections in Somali women and Somali women themselves express both a preference for vaginal birth and fear about undergoing caesarean section^8^–^11^,^18^ then the raised rate of caesarean section in Somali-born women after resettlement is concerning. This is particularly so in a community where large families are the desired norm.^18^ Three studies have suggested that caesarean section may be resorted to when care providers are inexperienced in caring appropriately for infibulated women to enable them to give birth vaginally.^11^–^13^ Given that the majority of Somali-born women are likely to have undergone traditional genital cutting and infibulation in Somalia during childhood then investigation of current clinical practice in pregnancy and delivery care for Somali women giving birth postmigration would appear warranted. Other issues may also be affecting caesarean section rates for Somali women, including language difficulties in communicating with care providers.

The infant outcomes reported here show a mixed picture largely consistent with findings in previous studies. Somali-born women were not more likely to give birth preterm or to give birth to infants of low birthweight compared with receiving country-born women. Yet in most—although not all—countries, Somali-born women *were* more likely to have infants with poor 5-minute Apgars. Explanations for the poorer condition of Somali infants are likely to be many and unfortunately this study has been unable to explore them. Raised rates of stillbirth in Somali-born women have also been found in previous studies.^1^,^2^,^6^ Whether these are amenable to intervention will remain unclear until further research elucidates the contributing factors. Although Somali-born women in our study did not have statistically significantly higher rates of early neonatal or late neonatal deaths compared with receiving country-born women, the point estimates were higher in both Norway and Sweden, although not in Canada (Ontario) or in Australia (Victoria).

More detailed investigation and analysis than is possible with these data sets is needed to study causes of deaths, and particularly any potentially avoidable deaths among infants of Somali-born women. One such previous study from Sweden, although small, reported a range of factors found to be more often associated with perinatal deaths among Somali-born women than in the deaths to Swedish-born mothers.^19^ These included: insufficient surveillance of intrauterine growth restriction, inadequate medication, misinterpretation of cardiotocography, delay in seeking health care and refusal of emergency caesarean section by mothers, and miscommunication because of lack of interpretation.

Interventions aimed at improving maternity care and outcomes for resettled Somali women appear to be rare, with just one study found. This describes a doula support program for Somali women, started in 2005 in the USA.^20^ A total of 123 Somali women gave birth with the support in labour of a trained Somali doula. Comprehensive outcome data are not reported, but the proportions of women who had a caesarean section in the doula-supported group were lower compared with the 165 Somali women who had given birth during the same time period, at the same hospital, but without a doula: 17 versus 26%; and for women having first births the proportions were 18 and 28%, respectively. Women involved in the program expressed a great deal of satisfaction with doula support, as did the nurses caring for doula-supported women. These findings need cautious interpretation given that the intervention was not evaluated within a randomised trial and the numbers involved were not large, but they suggest that further investigation of this type of labour support and assistance with communication may be worthwhile.

## Conclusion

Together with previous research, this study raises issues to be addressed in the provision of maternity care for resettled Somali women. There is a clear need for implementation and careful evaluation of strategies to improve communication and understanding between Somali women and their care providers, to ensure that care in pregnancy and at birth is appropriate to women’s needs and circumstances, and to improve the pregnancy outcomes of Somali women.

## Disclosure of interests

We declare that we have no conflicts of interest.

## Contribution to authorship

R.S. initiated and co-ordinated the study, analysed the data and drafted the manuscript. All other authors were involved in data provision and in the critical revision and finalisation of the manuscript.

## Details of ethics approval

Approval for the secondary analysis of the routine data used in this study is held by the individual authors who provided data.

## Funding

No funding was sought or obtained to undertake this specific study. Individual authors were supported through their institutional or grant funded employment to participate. No funding source had any role in the conduct, analysis or interpretation of the study.
